# Supplementary material for: Brain-to-cervical lymph node signaling after stroke
Source: Nat Commun. 2019 Nov 22;10:5306. doi: 10.1038/s41467-019-13324-w (PMC6876639; doi:10.1038/s41467-019-13324-w)
Supplement: Supplementary file 1 — Supplementary Information [file 41467_2019_13324_MOESM1_ESM.pdf]

Supplementary information

Brain-to-cervical lymph node signaling after stroke

Esposito et al.

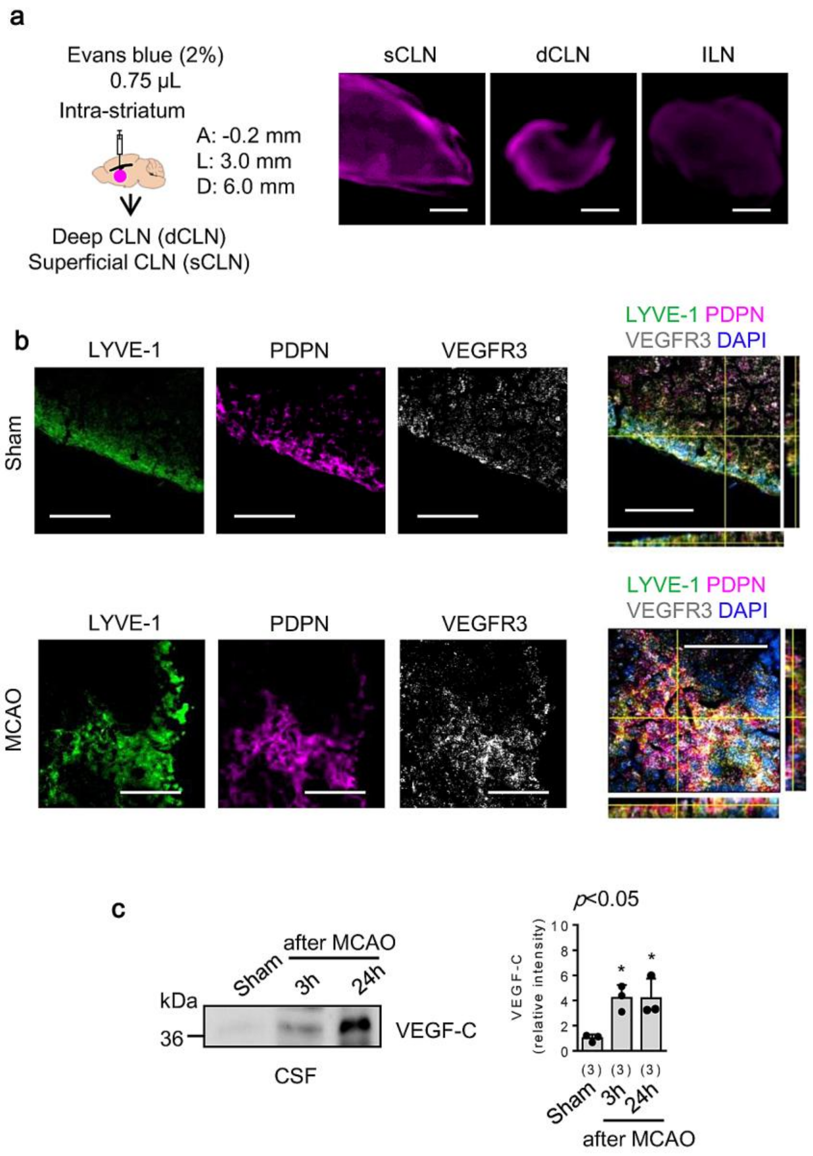

Supplementary figure 1. The brain-to-cervical lymph node signaling involving VEGF-C/VEGFR3 pathway after cerebral ischemia in rats:

a. Evans Blue dye (2%, 0.75 μL) was injected into intra-striatum (Anterior from bregma; -0.2 mm, Lateral from bregma; 3.0 mm, Depth; 6.0 mm) in normal male Sprague Dawley (SD) rats. Evans Blue fluorescence was detected in cervical lymph nodes (CLNs) but not in inguinal lymph nodes (ILNs) at 3 hours after intra-striatum injection. **b.** Male SD rats were subjected to 100 min transient focal cerebral ischemia. Confocal microscopy confirmed co-localization of LYVE-1, Podoplanin (PDPN), and VEGFR3 in sham or MCAo. **c.** VEGF-C was rapidly increased in cerebrospinal fluid after focal cerebral ischemia (n=3 biologically independent animals). *P<0.05 vs Sham, one-way ANOVA followed by Fisher's LSD test. All values are mean +/- S.D.

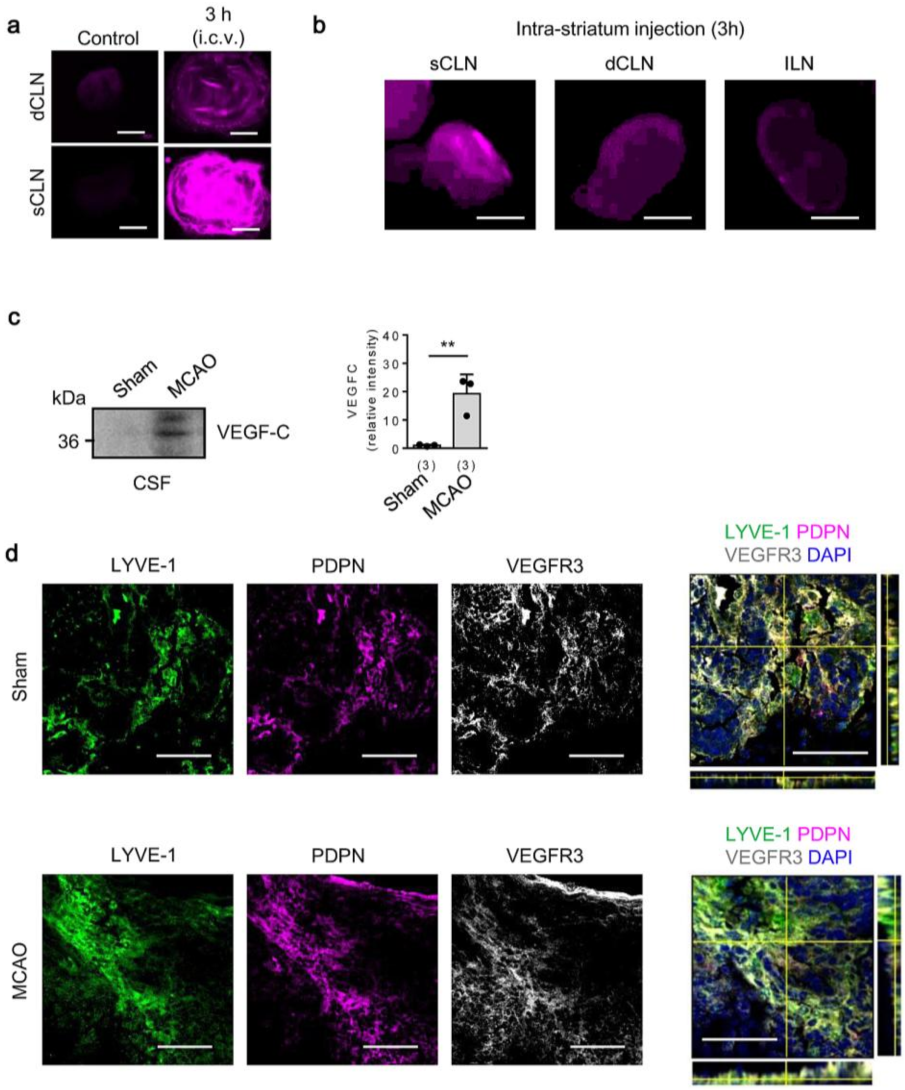

Supplementary figure 2. The brain-to-cervical lymph node signaling involving VEGF-C/VEGFR3 pathway after cerebral ischemia in mice:

a. Evans Blue dye (2%, 5 µL) was injected into lateral ventricles (Anterior from bregma; -0.5 mm, Lateral from bregma; 0.8 mm, Depth; 2.5 mm) in normal male C57BL6 mice. Evans Blue fluorescence was detected in CLNs but not in ILNs after i.c.v. **b.** Evans Blue dye (2%, 0.2 µL) was injected into striatum (Anterior from bregma; 0 mm, Lateral from bregma; 2.0 mm, Depth; 3.5 mm) in normal male C57BL6 mice. Evans Blue fluorescence was detected in superficial CLNs. **c.** Male C57BL6 mice were subjected to 60 min middle cerebral artery occlusion. Western blot confirmed that VEGF-C was increased in CSF at 24 hours after focal cerebral ischemia in mice (n=3 biologically independent animals). ***P*<0.01, unpaired t-test. All values are mean +/- S.D. **d.** Confocal imaging confirmed co-localization of LYVE-1, Podoplanin (PDPN), and VEGFR3.

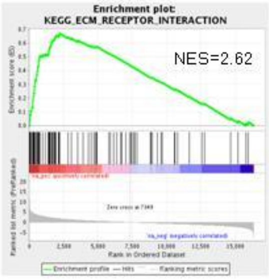

rank	gene
1	THBS1
2	ITGA3
3	ITGB4
4	COL4A1
5	LAMB2
6	FN1
7	COL4A2
8	HSPG2
9	ITGA1
10	ITGB5

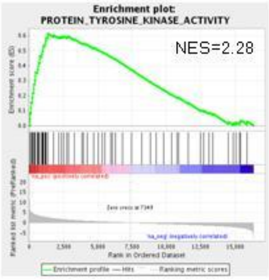

rank	gene
1	TIE1
2	FLT1
3	PDGFRA
4	CRIM1
5	MET
6	TEK
7	FGFR1
8	RYK
9	INSR
10	ROR1

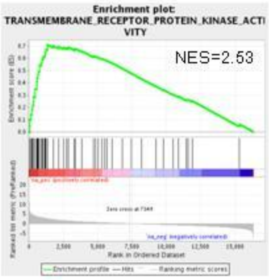

rank	gene
1	TIE1
2	FLT1
3	PDGFRA
4	CRIM1
5	MET
6	TEK
7	FGFR1
8	RYK
9	INSR
10	ACVRL1

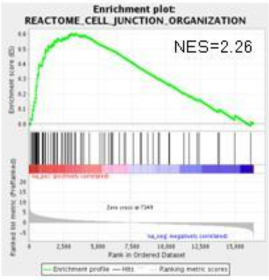

rank	gene
1	ITGB4
2	CDH5
3	PARVA
4	F11R
5	CTNND1
6	PARVB
7	CADM1
8	CD151
9	LAMA3
10	PARD3

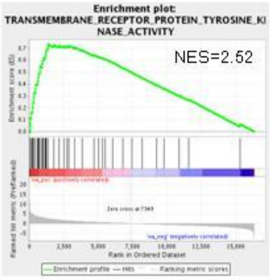

rank	gene
1	TIE1
2	FLT1
3	PDGFRA
4	CRIM1
5	MET
6	TEK
7	FGFR1
8	RYK
9	INSR
10	ROR1

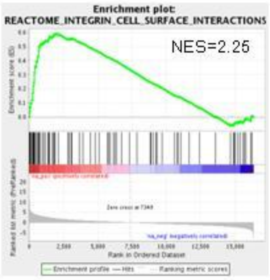

rank	gene
1	THBS1
2	ITGA3
3	ITGB4
4	COL4A1
5	LAMB2
6	FN1
7	COL4A2
8	JAM2
9	F11R
10	ITGA1

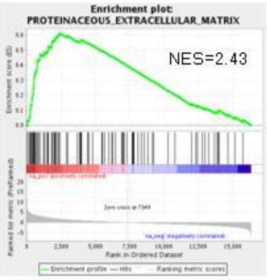

rank	gene
1	ADAMTS9
2	MGP
3	CTGF
4	LAMB2
5	COL4A2
6	LUM
7	ODAM
8	ECM1
9	COL15A1
10	FBLN2

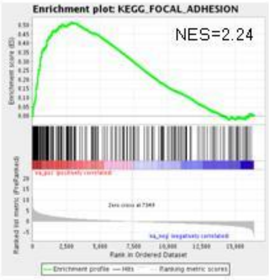

rank	gene
1	THBS1
2	ITGA3
3	FLT1
4	ITGB4
5	PDGFRA
6	COL4A1
7	EGF
8	PARVA
9	LAMB2
10	FN1

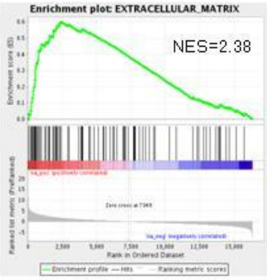

rank	gene
1	ADAMTS9
2	MGP
3	CTGF
4	LAMB2
5	COL4A2
6	LUM
7	ODAM
8	ECM1
9	COL15A1
10	FBLN2

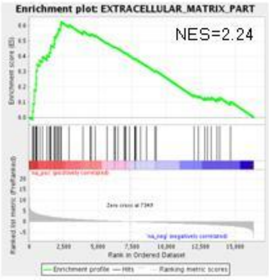

rank	gene
1	LAMB2
2	COL4A2
3	LUM
4	ODAM
5	COL15A1
6	LAMA3
7	LAMB1
8	LAMA4
9	COL6A3
10	COL1A2

Supplementary figure 3. GSEA enrichment plot: Normalized Enrichment Score (NES) was obtained from the ratio of the observed ES to the mean ES computed across 1000 random distributions of the members of the gene set across the ranked list. Gene Set Enrichment Analysis (GSEA) suggested that after cerebral ischemia, differentially expressed genes were largely related to matrix pathways and transmembrane receptor protein tyrosine kinase activity.

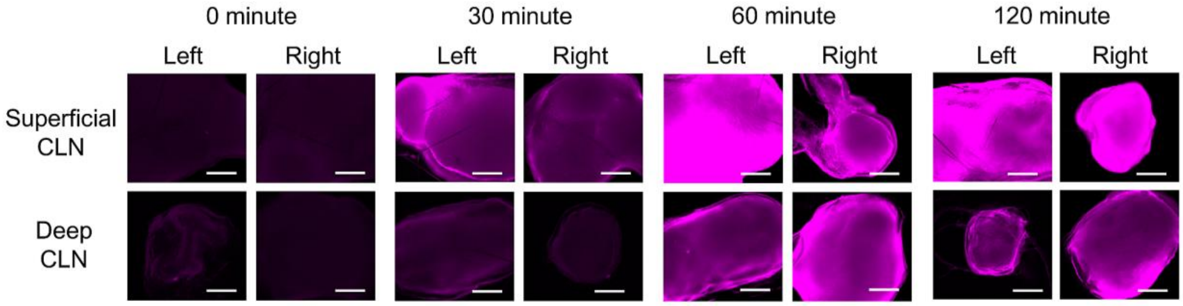

Supplementary figure 4. Intranasal cavity injection to target cervical lymph nodes:
Evans Blue (10 µL, 2%) was injected into nasal cavity in normal male C57BL6 mice. Within 60 min, Evans Blue-derived fluorescence was detected in both superficial and deep CLNs.

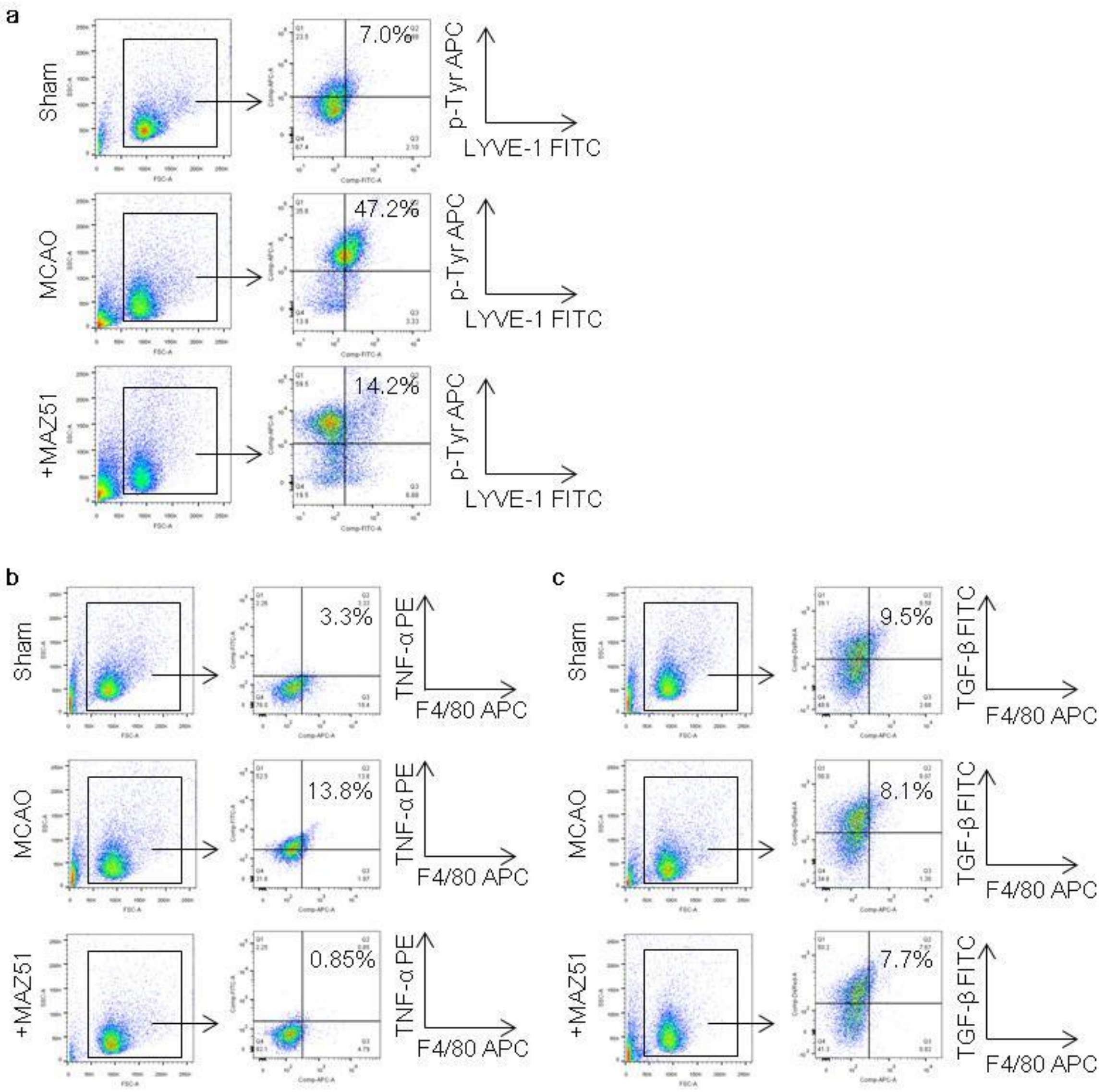

Supplementary figure 5. FACS analysis in superficial CLNs after focal ischemia in mice:

a-c. Mice were subjected to transient 60 min focal ischemia and MAZ51 (3 ng/10 µL) was injected into nasal cavity right after reperfusion. FACS analysis demonstrated that MAZ51 treatment significantly decreased lymphatic vessel **(a)** phosphorylation, **(b)** pro-inflammatory macrophages in CLNs, while **(c)** TGF-β positive macrophages were unchanged by the treatment at 72 hours after focal cerebral ischemia. **(a)** gating panels correspond to Fig. 4b, **(b)** and **(c)** gating panels correspond to Fig. 4c.

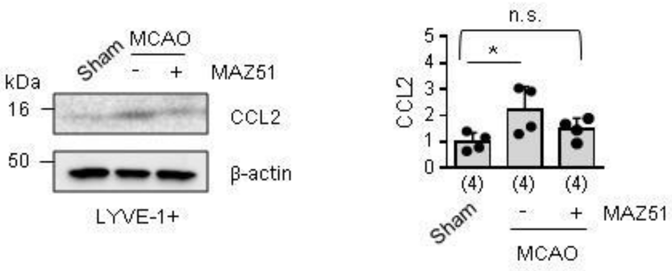

Supplementary figure 6. Blockade of VEGFR3 tyrosine kinase reduced CCL2 inflammatory chemokine: Mice were subjected to transient 60 min focal ischemia and vehicle (PBS 10 µL) or MAZ51 (3 ng/10 µL) was injected into nasal cavity right after reperfusion. Western blot confirmed that CCL2 was significantly increased at 24 hours in post-stroke superficial CLN and MAZ51 treatment reduced CCL2 expression (n=4 biologically independent animals). **P*<0.05, one-way ANOVA followed by Fisher's LSD test. All values are mean +/- S.D.

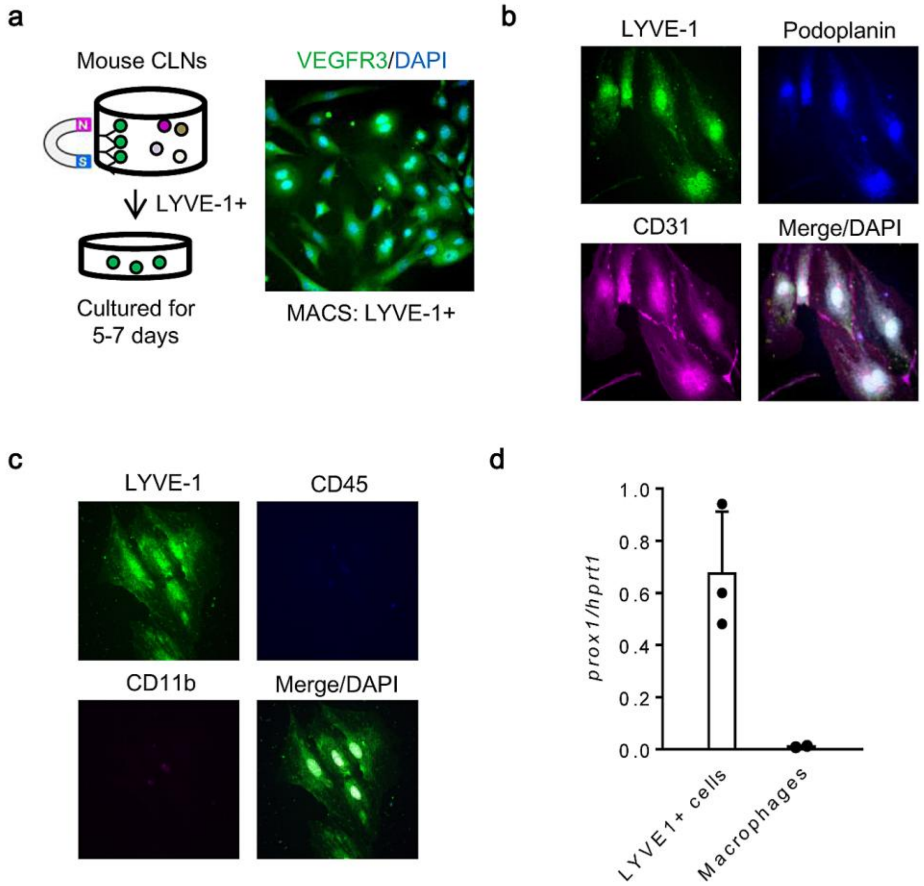

Supplementary figure 7. Lymphatic endothelial isolation from CLNs:

a. LYVE-1 antibody-conjugated magnetic beads used for lymphatic endothelial isolation using CLNs isolated from 4 mice. After culturing for 7 days, immunostaining showed that isolated cells expressed VEGFR3. **b, c.** Immunocytochemistry defined that LYVE-1 positive cells expressed Podoplanin and CD31, but they did not express CD45 and CD11b. **d.** qPCR analysis showed that LYVE-1 positive cells highly expressed a lymphatic vessel marker, prox1 (n=3 biologically independent samples). Peritoneal macrophages isolated from mice did not express prox1 (n=2 biologically independent samples). All values are mean +/- SD.

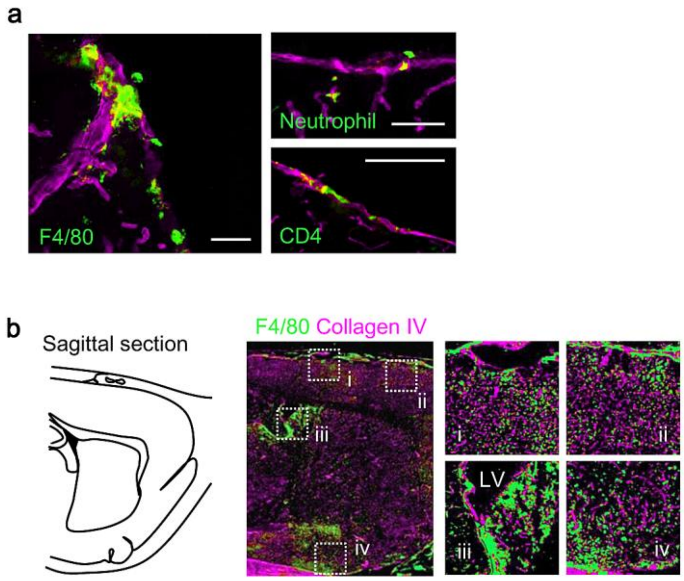

Supplementary figure 8. Immune cell accumulation near leptomeninges after stroke:

a. Immune cells were found in close proximity to leptomeninges in the ipsilateral hemisphere. Red: Collagen IV. Note: Collagens including Collagen IV are the most abundant ECM structures in meninges. **b.** Further staining in sagittal brain section confirmed that immune cells were accumulated in near leptomeninges (region i, ii, iv), subventricular zone (region iii), and brain parenchyma.

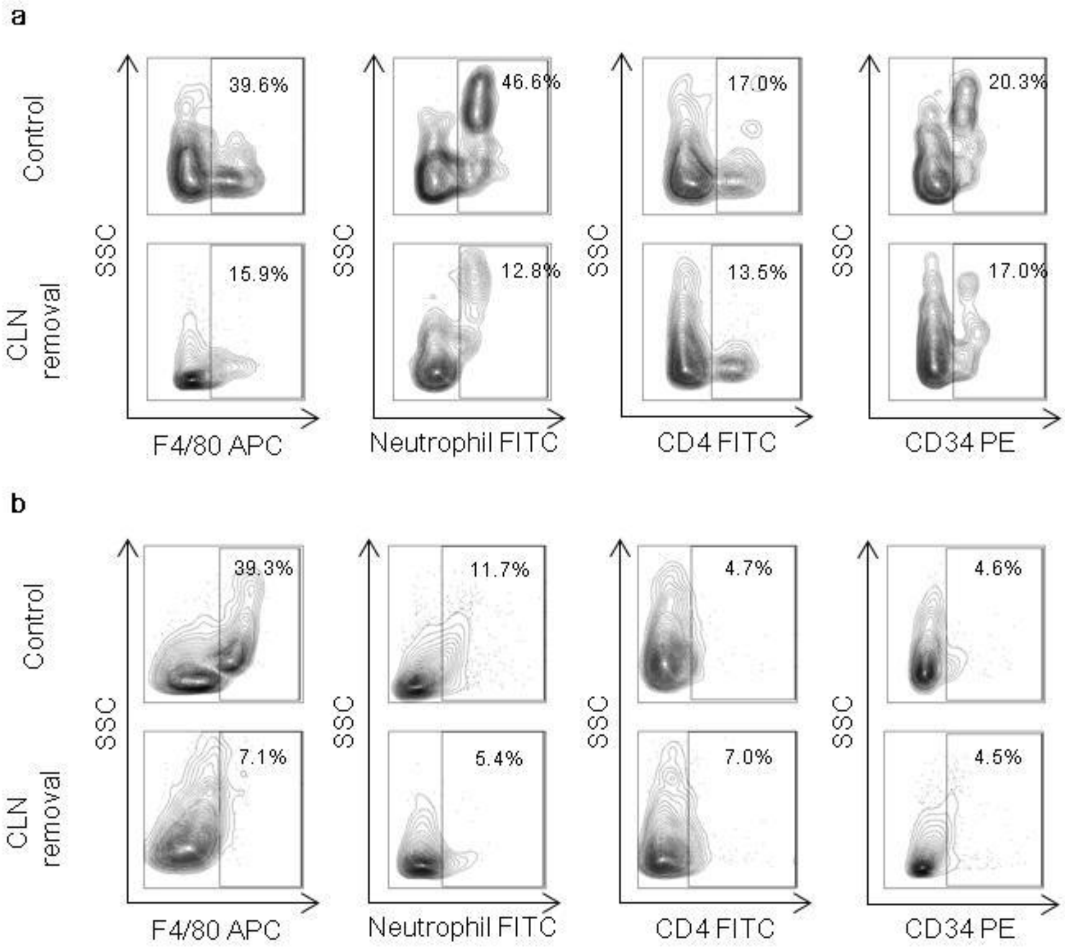

Supplementary figure 9. FACS analysis in blood and cerebral cortex after focal ischemia in mice: Gating strategy in flow cytometry analysis in **(a)** blood and **(b)** cerebral cortex after stroke. **(a)** gating panels correspond to **Fig. 7b**, **(b)** gating panels correspond to **Fig. 7c.**

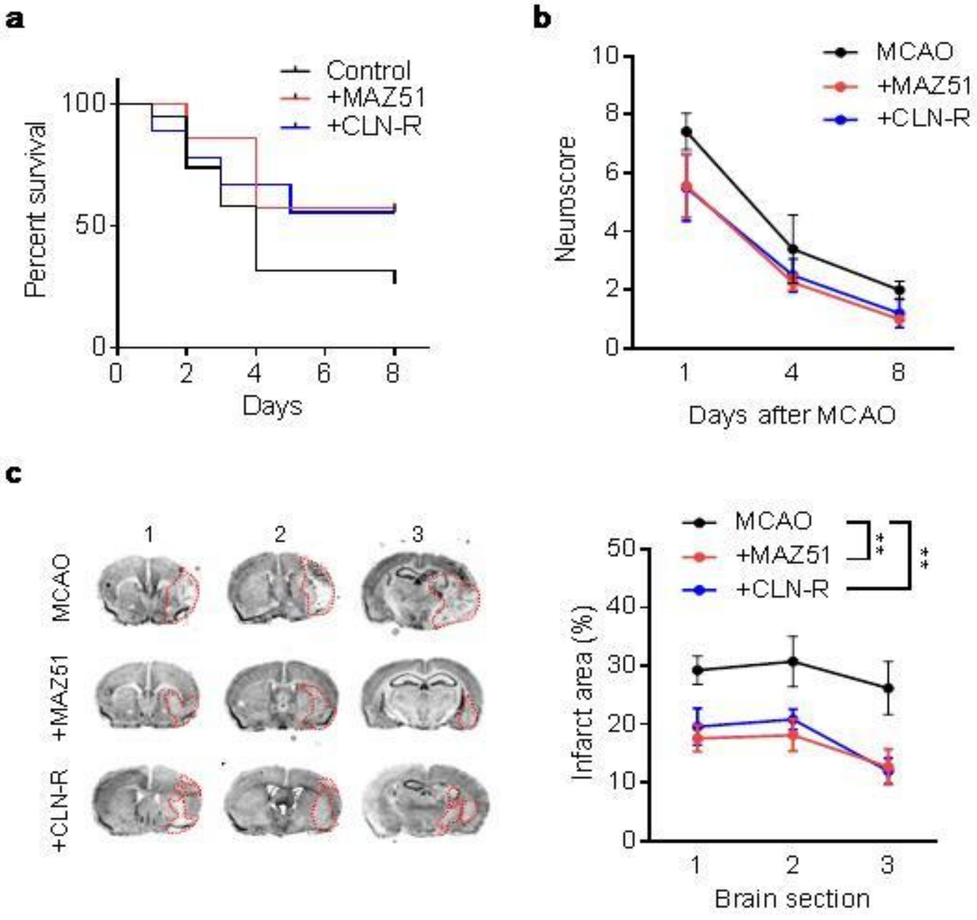

Supplementary figure 10. Neurological outcomes after blockade of VEGFR3 or CLN lymphadenectomy: **a.** Treatment with MAZ51 or CLN lymphadenectomy improved survival rate after stroke. **b.** Neuroscore were slightly better in MAZ51 or CLN removal group compared to non-treatment group, but there was no statistic significance. Repeated two-way ANOVA. **c**. Nissl staining at day 8 after focal cerebral ischemia confirmed that MAZ51 or CLN lymphadenectomy group showed smaller infarct size compared to non-treatment group (Red dotted line: infarct area, MCAO; n=9 biologically independent animals, +MAZ51; n=8 biologically independent animals, CLN-R; n=7 biologically independent animals). *P<0.05, one-way ANOVA followed by Fisher's LSD test. All values are mean +/- SD.
