# Supplementary material for: Brain-to-cervical lymph node signaling after stroke
Source: Nat Commun. 2019 Nov 22;10:5306. doi: 10.1038/s41467-019-13324-w (PMC6876639; doi:10.1038/s41467-019-13324-w)
Supplement: Supplementary file 3 — Reporting Summary [file 41467_2019_13324_MOESM3_ESM.pdf]

nature research

Corresponding author(s): Kazuhide Hayakawa, Eng H. Lo

# Reporting Summary

Nature Research wishes to improve the reproducibility of the work that we publish. This form provides structure for consistency and transparency in reporting. For further information on Nature Research policies, see Authors & Referees and the Editorial Policy Checklist.

## Statistical parameters

When statistical analyses are reported, confirm that the following items are present in the relevant location (e.g. figure legend, table legend, main text, or Methods section).

| n/a | Confirmed | |
|---|---|---|
| ☐ | ☒ | The exact sample size ($n$) for each experimental group/condition, given as a discrete number and unit of measurement |
| ☐ | ☒ | An indication of whether measurements were taken from distinct samples or whether the same sample was measured repeatedly |
| ☐ | ☒ | The statistical test(s) used AND whether they are one- or two-sided<br>*Only common tests should be described solely by name; describe more complex techniques in the Methods section.* |
| ☐ | ☒ | A description of all covariates tested |
| ☐ | ☒ | A description of any assumptions or corrections, such as tests of normality and adjustment for multiple comparisons |
| ☐ | ☒ | A full description of the statistics including central tendency (e.g. means) or other basic estimates (e.g. regression coefficient) AND variation (e.g. standard deviation) or associated estimates of uncertainty (e.g. confidence intervals) |
| ☐ | ☒ | For null hypothesis testing, the test statistic (e.g. $F$, $t$, $r$) with confidence intervals, effect sizes, degrees of freedom and $P$ value noted<br>*Give P values as exact values whenever suitable.* |
| ☒ | ☐ | For Bayesian analysis, information on the choice of priors and Markov chain Monte Carlo settings |
| ☒ | ☐ | For hierarchical and complex designs, identification of the appropriate level for tests and full reporting of outcomes |
| ☐ | ☒ | Estimates of effect sizes (e.g. Cohen's $d$, Pearson's $r$), indicating how they were calculated |
| ☐ | ☒ | Clearly defined error bars<br>*State explicitly what error bars represent (e.g. SD, SE, CI)* |

*Our web collection on statistics for biologists may be useful.*

## Software and code

Policy information about availability of computer code

| | |
|---|---|
| Data collection | No softwares were used to collect data. |
| Data analysis | Infarction volumes were quantified on TTC stained sections using the “indirect” morphometric method (Lin et al., 1993) with Image J. For wester blot analysis optical density was assessed using the NIH Image analysis software software. Statistical analysis was performed with GraphPad Prism 6.01 |

For manuscripts utilizing custom algorithms or software that are central to the research but not yet described in published literature, software must be made available to editors/reviewers upon request. We strongly encourage code deposition in a community repository (e.g. GitHub). See the Nature Research guidelines for submitting code & software for further information.

## Data

Policy information about availability of data

All manuscripts must include a data availability statement. This statement should provide the following information, where applicable:

- Accession codes, unique identifiers, or web links for publicly available datasets
- A list of figures that have associated raw data
- A description of any restrictions on data availability

The authors declare that the data supporting the findings of this study are available within the paper and its supplementary information files or from the corresponding author upon reasonable request.

# Field-specific reporting

Please select the best fit for your research. If you are not sure, read the appropriate sections before making your selection.

☒ Life sciences ☐ Behavioural & social sciences ☐ Ecological, evolutionary & environmental sciences

For a reference copy of the document with all sections, see nature.com/authors/policies/ReportingSummary-flat.pdf

# Life sciences study design

All studies must disclose on these points even when the disclosure is negative.

| | |
|---|---|
| Sample size | Sample size was predetermined using the software available online: https://www.danielsoper.com/statcalc/calculator.aspx?id=47 The calculation was based on Cohen's d value where SD and average were estimated from our historical and preliminary data. |
| Data exclusions | For in vivo studies animals that did not demonstrate a significant reduction to less than 30% baseline LDF values during MCAO , or rapid restoration of the LDF signal during reperfusion were excluded. |
| Replication | For in vitro experiments, each experiment was repeated at least 3 times. Details are in the method section |
| Randomization | The samples/animals were randomly assigned (simple randomization with computer-generated random numbers), before starting the experiments. |
| Blinding | All procedures and measurements were performed in a blinded and randomized fashion. |

# Reporting for specific materials, systems and methods

### Materials & experimental systems

| n/a | Involved in the study |
|---|---|
| ☒ | ☐ Unique biological materials |
| ☐ | ☒ Antibodies |
| ☒ | ☐ Eukaryotic cell lines |
| ☒ | ☐ Palaeontology |
| ☐ | ☒ Animals and other organisms |
| ☒ | ☐ Human research participants |

### Methods

| n/a | Involved in the study |
|---|---|
| ☒ | ☐ ChIP-seq |
| ☐ | ☒ Flow cytometry |
| ☒ | ☐ MRI-based neuroimaging |

## Antibodies

| | |
|---|---|
| Antibodies used | anti-β-actin (1:1,000, A5441, Sigma-aldrich), anti-p-Tyr antibody (1:500, sc-7020, Santa Cruz), anti-VEGF-C antibody (1:500, sc-374628, Santa Cruz), anti-VEGFR3 antibody (1:500, sc-365748, Santa Cruz), anti-iNOS antibody (1:500, ab3523, Abcam), anti-IL-1β antibody (1:500, ab9722, Abcam), anti-TNF-α antibody (1:200, GTX110520, GeneTex), anti-TGF-β antibody (1:200, ab64715, Abcam), anti-CCL28 antibody (1:500, MAB717, R&D systems), anti-Podoplanin antibody (1:200, sc-166906, Santa Cruz), anti-CD31 antibody (1:200, 550274, BD biosciences), anti-LYVE-1 antibody (1:500, NB100-725B, NOVUS biologicals), anti-vWF antibody (1:100, A0082, Agilent), FITC anti-CD4 antibody (1:100, 561828, BD Biosciences), FITC Neutrophil antibody (1:100, ab53453, Abcam), PE anti-CD34 antibody (1:100, 551387, BD Biosciences), APC anti-F4/80 antibody (1:200, 123116, BioLegend), FITC anti-CD16/32 antibody (1:200, 101306, BioLegend), PE anti-CD11b antibody (1:200, 557397, BD biosciences), anti-CD45 antibody (1:400, 20103-1-AP, Proteintech). |

Validation | Antibodies used in this study were validated by comparing with the no-primary and no-secondary controls.

## Animals and other organisms

Policy information about studies involving animals; ARRIVE guidelines recommended for reporting animal research

| | |
|---|---|
| Laboratory animals | All experiments were performed following an institutionally approved protocol in accordance with National Institutes of Health guidelines and with the United States Public Health Service's Policy on Human Care and Use of Laboratory Animals and following Animals in Research: Reporting In vivo Experiments (ARRIVE) guidelines. Male Sprague-Dawley rats (320 to 340 g) and male C57BL/6 male mice (23 to 27 g) were used in this study. |
| Wild animals | No wild animals were used in this study. |
| Field-collected samples | No field-collected were used in this study. |

## Flow Cytometry

### Plots

Confirm that:

☒ The axis labels state the marker and fluorochrome used (e.g. CD4-FITC).

☒ The axis scales are clearly visible. Include numbers along axes only for bottom left plot of group (a 'group' is an analysis of identical markers).

☒ All plots are contour plots with outliers or pseudocolor plots.

☒ A numerical value for number of cells or percentage (with statistics) is provided.

### Methodology

| | |
|---|---|
| Sample preparation | Tissues collected from leptomeninges, and cervical lymph nodes are gently minced and then digested at 37°C for 30 min with an enzyme cocktail. |
| Instrument | BD Fortessa |
| Software | FACS data were analyzed by FlowJo version 10. |
| Cell population abundance | In this study, percentage of our target population was 10-30%. When inducing loss-of-function or under unstimulated conditions, these population had 1-10%. |
| Gating strategy | FACS analysis was performed using an unstained or phenotype control for determining appropriate gates, voltages, and compensations required in multivariate flow cytometry. |

☒ Tick this box to confirm that a figure exemplifying the gating strategy is provided in the Supplementary Information.
